# A High Performance Lithium-Ion Capacitor with Both Electrodes Prepared from Sri Lanka Graphite Ore

**DOI:** 10.3390/ma10040414

**Published:** 2017-04-14

**Authors:** Xiaoyu Gao, Changzhen Zhan, Xiaoliang Yu, Qinghua Liang, Ruitao Lv, Guosheng Gai, Wanci Shen, Feiyu Kang, Zheng-Hong Huang

**Affiliations:** State Key Laboratory of New Ceramics and Fine Processing, School of Materials Science and Engineering, Tsinghua University, Beijing 100084, China; xiaoyu_ustb@163.com (X.G.); zhanchangzhen@foxmail.com (C.Z.); yuxiaoliang2412@hotmail.com (X.Y.); lqingh506@163.com (Q.L.); lvruitao@tsinghua.edu.cn (R.L.); gaigs@tsinghua.edu.cn (G.G.); shenwc@mail.tsinghua.edu.cn (W.S.); fykang@tsinghua.edu.cn (F.K.)

**Keywords:** lithium-ion capacitor, Sri Lanka graphite, graphitic porous carbon, KOH activation

## Abstract

The natural Sri Lanka graphite (vein graphite) is widely-used as anode material for lithium-ion batteries (LIBs), due to its high crystallinity and low cost. In this work, graphitic porous carbon (GPC) and high-purity vein graphite (PVG) were prepared from Sri Lanka graphite ore by KOH activation, and high temperature purification, respectively. Furthermore, a lithium-ion capacitor (LIC) is fabricated with GPC as cathode, and PVG as anode. The assembled GPC//PVG LIC shows a notable electrochemical performance with a maximum energy density of 86 W·h·kg^−1^ at 150 W·kg^−1^, and 48 W·h·kg^−1^ at a high-power density of 7.4 kW·kg^−1^. This high-performance LIC based on PVG and GPC is believed to be promising for practical applications, due to its low-cost raw materials and industrially feasible production.

## 1. Introduction

With the rapid development of the world’s economy and continuous consumption of fossil energy, electrochemical energy storage devices are in urgent demand for the application of clean energy and electric vehicles [1,2]. In particular, electrochemical capacitors and lithium ion batteries (LIBs) dominate the energy supply for portable electronics. Electrochemical capacitors can be classified into three main groups: electrical double-layer capacitors (EDLC); pseudo-capacitors; and hybrid/asymmetric capacitors [3]. During the past few years, considerable attention has been paid to lithium-ion capacitors (LICs) which are hybrid devices composed of LIBs-type anode and supercapacitors (SCs)-type cathode [4,5,6,7,8]. It is well-known that LIBs, compared to SCs, possess a relatively high energy density, but limited power density and poor cycle performance, because of their different energy storage mechanism. Thus, LICs are intended to combine the advantages of both LIBs and SCs by the synergetic effect of faradic intercalation/de-intercalation reaction, and non-faradic electrochemical double layer adsorption/desorption behavior.

Accordingly, the electrode materials play a crucial role in the electrochemical performance of LICs. Energy density can be greatly influenced by the working voltage window of hybrid devices and specific capacity of LIBs component [9,10]. Further, power density mostly depends on the rate performance of faradic battery-type anode and electric conductivity of supercapacitor-type cathode [11]. Therefore, extensive studies have been undertaken to prepare excellent electrode materials. As for the battery-type anode of LICs, various intercalation materials have been discussed, such as carbonaceous materials [12,13,14,15,16], titanium based compounds [7,17], and manganese oxides [18,19]. Among these compounds, graphite is still widely and commercially used in LICs because of its low lithium-ion intercalation potential (near 0.1 V vs. Li^+^/Li), high theory capacity (372 mAh·g^−1^ for LiC_6_) and low cost. It has been reported that industrially feasible synthesis of high-quality graphite can be achieved by high temperature purification [20].

As for the cathode, porous carbon is a promising candidate due to its large specific area and chemical stability. However, the poor electrical conductivity results in a limited power capacity of the hybrid system. In order to obtain porous carbon with an excellent conductivity, introducing conductive structures is an attractive approach [11,21,22,23]. Ji et al. discussed various graphene-based nanocomposites synthesized as nanostructured electrodes for electrochemical capacitors. Despite the special properties of graphene, the performance of graphene-based capacitors can be further enhanced by incorporating secondary carbon phase to improve electrolyte–electrode accessibility and electrical conductivity of electrode [3]. Yu et al. [9] designed an advanced carbonaceous cathode material by integrating mesoporous carbon nanospheres into graphene nanosheets with a further nitrogen doping treatment. The obtained nanocomposite possessed an effective conducting network, which greatly facilitated the electron transportation. Analogously, three dimensional porous graphene macroform were coupled with anode of Li_4_T_5_O_12_ to fabricate a hybrid capacitor which delivers a maximum energy density of 72 W·h·kg^−1^ at 650 W·kg^−1^, and 40 W·h·kg^−1^ at a high power density of 8.3 kW·kg^−1^ [24]. The ethylene glycol assisted-hydrothermally reduced graphene delivered a superior power density used as cathode material for the LICs [25]. Based on the above results, we infer that the design of cathode materials is of great importance in achieving a high-power performance of the LICs. However, most studies were focused on the enhancement of electrochemical performance. The preparation of such electrode materials on a large-scale, with low-cost, is limited. The easy manufacturing process of electrodes is in urgent demand, but still quite challenging for LICs.

In this work, we developed a facile two-step route to prepare both electrodes of LICs. Starting from the low-cost Sri Lanka graphite ore, vein graphite (VG) and high-purity vein graphite (PVG) were produced by chemical purification and high temperature purification, respectively. VG sample was further activated by potassium hydroxide to produce partially-graphitized porous carbon (designated as GPC), and employed as a cathode of LIC. PVG was pre-lithiated and served as anode for LIC. All the aforementioned procedures can be performed on a large scale. The integrated hybrid system delivers a high-energy density of 86 W·h·kg^−1^ at 150 W·kg^−1^, and maintains 48 W·h·kg^−1^ at a high-power density of 7.4 kW·kg^−1^.

## 2. Materials and Methods

### 2.1. Synthesis of the GPC

GPC was prepared by chemical activation of VG. Typically, VG was mixed with potassium hydroxide with a mass ratio of 1:4. VG was added into KOH solution and dispersed by ultrasonication. The resultant homogeneous slurry was dried at 80 °C, the product was then placed into the tube furnace and calcined at 800 °C for one hour, followed by calcination at 950 °C for 1 h, with a heating rate of 5 °C·min^−1^ in argon atmosphere. After the activation process, the product was sequentially washed with diluted hydrochloric acid solution and deionized water, until the PH value of filtrate became neutral. Finally, pure GPC sample was obtained after drying at 120 °C.

### 2.2. Characterization

The morphology and microstructure of materials were observed by using a scanning electron microscopy (SEM, Hitachi S-5500, Tokyo, Japan) and transmission electron microscopy (TEM, JEM-2010F, JEOL Ltd., Tokyo, Japan). X-ray diffraction (XRD) patterns and Raman spectra were examined by using Bruker-D8 advance spectrometer (Bruker, Germany) with Cu Kα radiation (*λ* = 0.154056 nm), and LabRAM HR Evolution Raman spectrometer (HORIBA Jobin Yvon, Paris, France) with a 532 nm excitation laser, respectively. N_2_ adsorption-desorption isotherms were measured using a Quantachrome adsorb volumetric sorption apparatus (Quantachrome Instruments, Boynton Beach, FL, USA). The specific surface area was calculated by the Brunauer-Emmett-Teller (BET) method, and pore size distribution was obtained by the density functional theory (DFT) method.

### 2.3. Electrochemical Measurements

All the electrochemical performances were measured by assembling 2032 coin cells. For cathode materials, the porous carbons (GPC or commercially available activated carbon YP-17D) were mixed with PVDF and carbon black (Super P), at a mass ratio of 75:15:10 in *N*-Methyl-2-pyrrolidone solvent to form homogeneous slurries. This was then coated onto aluminum foils. For anode materials, PVG was mixed with PVDF and Super P at a mass ratio of 80:10:10, and the mixed slurry was then coated onto copper foils. The foils were dried at 80 °C for 4 h, and then at 120 °C in vacuum overnight. LIB half cells were assembled using a lithium metal electrode with PVG anode. Symmetric supercapacitors were assembled with YP-17D and GPC electrodes, respectively. LIC devices were assembled with porous carbon (GPC and YP-17D) as cathode and pre-lithiated PVG as anode at a mass ratio of 1.5:1. All devices mentioned above were fabricated in an argon-filled glovebox, and the electrolyte used was 1 M LiPF_6_ in EC/DEC (1:1 by volume).

LAND battery system was used to perform the rate capacity tests of the LIB with the voltage windows of 0.01–3 V. Arbin-BT2000 test station was used to test the galvanostatic charge/discharge cycling (GC) curves of symmetric supercapacitors, and hybrid LIC devices with the voltage window 0–2.7 V and 2–4 V, respectively. Cycle voltammetry (CV) tests were performed on a VSP-300 multichannel electrochemical station.

### 2.4. Pre-Lithiation of PVG

PVG was pre-lithiated to serve as anode electrode in LICs. It was pre-lithiated by three full charge/discharge cycles with the potential range of 0.01–3 V (vs. Li^+^/Li), followed by a discharging process until the potential dropped to 0.1 V. All the cycles were run at 0.1 C.

## 3. Results and Discussion

### 3.1. Structure and Morphology

**Figure 1:**
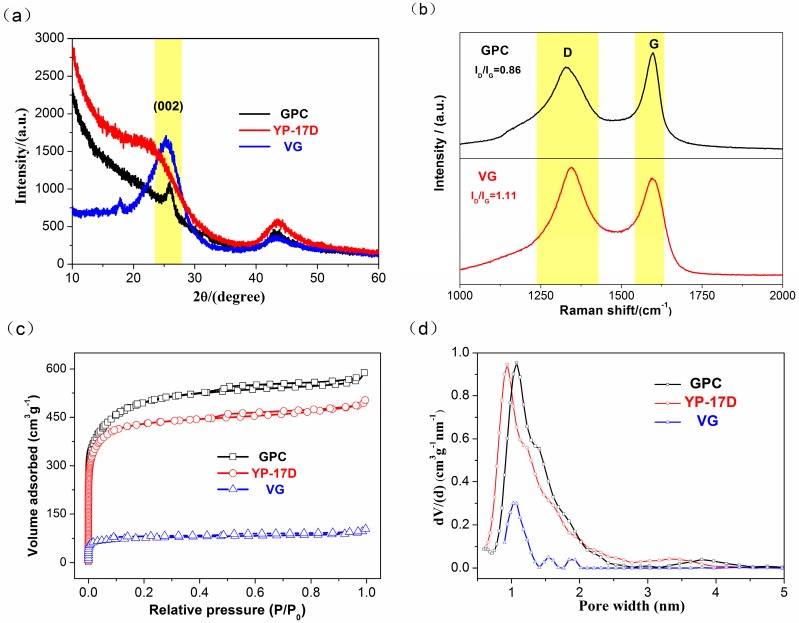
Microstructure analysis of three different samples. (**a**) X-ray diffraction (XRD) patterns of graphitic porous carbon (GPC), YP-17D and vein graphite (VG); (**b**) Raman spectra of VG and GPC; (**c**) N_2_ adsorption isotherms of GPC and YP-17D; (**d**) Pore size distribution curves of GPC, YP-17D and VG.

Figure 1a shows the XRD patterns of VG, GPC, and commercially-available activated carbon YP-17D. YP-17D reveals a broad peak at about 23°, corresponding to its amorphous characteristic. VG exhibits a broad peak centered at around ~25.5°, which is weaker than the typical (002) peak of graphite, but much stronger than that of amorphous YP-17D. This indicates the co-existence of a graphite-like structure and amorphous carbon, respectively [26]. In addition, another weak peak of VG was detected around 2θ = 18°, attributing to the presence of associated mineral impurities. In comparison with VG, GPC shows a broad peak associated with a satellite (002) peak, suggesting that GPC possesses considerate graphitic component dispersed in amorphous matrix after activation. Raman spectrum shown in Figure 1b was used to further study the structure of VG and GPC. Both the Raman spectra contain a D band at 1360 cm^−1^, and a G band at 1580 cm^−1^. Since the D and G bands were respectively ascribed to non-graphitic and graphitic structures, the intensity ratio of two peaks, (I_D_/I_G_) can be used to estimate the degree of graphitization for carbon materials. The smaller value of I_D_/I_G_ indicates a higher degree of graphitization. It can be seen that the value of I_D_/I_G_ decreases from 1.11 of VG to 0.86 of GPC, indicating A somewhat higher graphitization degree of GPC, which is in agreement with the XRD results. Since the amorphous carbon shows a higher reaction activity with KOH during high-temperature chemical activation than that of graphitized carbon [27,28], it can be deduced that during the KOH activation process, part of the amorphous carbon in VG was etched by KOH. Therefore, the GPC sample shows a higher degree of graphitization. 

The nitrogen adsorption/desorption isotherms of VG, GPC and YP-17D are shown in Figure 1c. All the curves exhibit type-I isotherm with a high adsorption in the low relative pressure, indicating dominant microporous structures. The specific surface area was determined by using the Brunauer-Emmett-Teller (BET) method. VG shows a smaller specific surface area of 326 m^2^·g^−1^. After the activation process, the GPC shows a greatly increased specific surface area of 1857 m^2^·g^−1^. While YP-17D displays an intermediate value of 1460 m^2^·g^−1^. The pore size distributions (PSD) were also calculated by the density functional theory (DFT) method, and the results are shown in Figure 1d. Both GPC and YP-17D reveal the co-existence of abundant micropores and small amount of mesopores. Further, the increase of microporosity was observed from VG to GPC. It can be deduced that GPC samples may be good candidates for supercapacitor electrode, due to their large specific surface area and favorable hierarchical porous structure.

**Figure 2:**
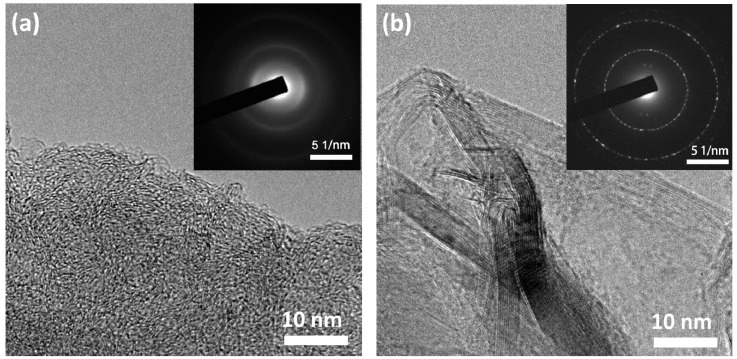
HRTEM image and inset selected area electron diffraction (SAED) pattern of (**a**) VG; (**b**) GPC.

Figure 2 shows the TEM images of VG and GPC. The selected area electron diffraction (SAED) pattern in the inset of Figure 2a displays obvious diffraction rings, which are characteristic of polycrystalline materials. It is worth noting that the HRTEM image shows no graphitic layers. The graphitic layers may be covered by thick amorphous carbon due to the presence of a large quantity of amorphous carbons in VG. From Figure 2b, it can be seen that GPC exhibits a mixture structure of ordered and disordered carbons, and the disordered sections are separated by random orientated and crisscrossed graphitic layers. Further, the inset image of Figure 2b shows bright diffraction rings and scattered diffraction spots, suggesting a higher graphitization degree of GPC compared to VG. The above results are in accordance with the characteristics of XRD and Raman patterns in Figure 1a,b. It is confirmed that both VG and GPC are composed of graphitic carbon and amorphous carbon. During the activation process, amorphous carbon was etched by KOH, thus graphitic carbon could be more easily detected in GPC. The abundant graphitic layers in GPC can improve electric conductivity, therefore GPC is expected to achieve a better high-rate capability as electrode material in SCs and LICs.

### 3.2. Electrochemical Performance of PVG in LIB

**Figure 3:**
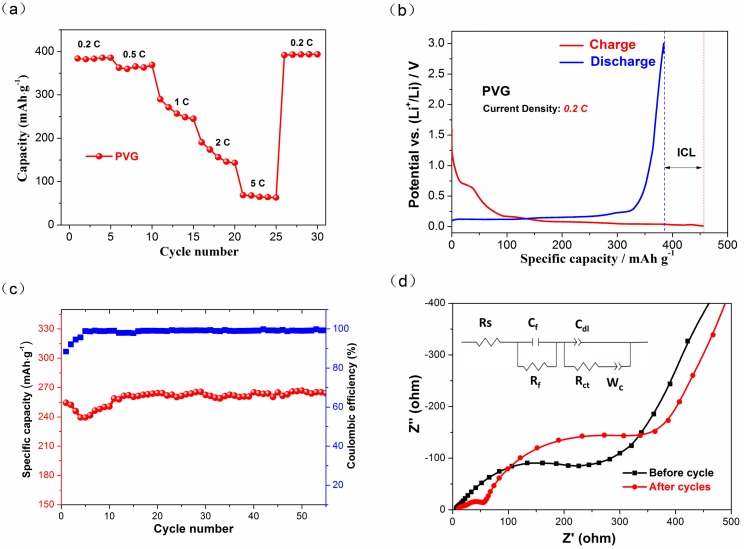
Electrochemical performance of the assembled high-purity vein graphite (PVG)/Li half-cell. (**a**) Rate performance; (**b**) The first galvanostatic charge-discharge profile at 0.2 C; (**c**) Cycle performance at 1 C; (**d**) The electrochemical impedance spectra (EIS) before and after cycles.

The rate performance and the first galvanostatic charge-discharge profile of PVG are showed in Figure 3a,b. The rate performance was tested at different current rates (0.2 C, 0.5 C, 1 C, 2 C and 5 C). Clearly, PVG reveals a relatively good rate performance and high capacity in Figure 3a. Its discharge capacity decreases from 384 mAh·g^−1^ to 170 mAh·g^−1^, with almost 43% capacity retention when the rate increases from 0.2 C to 2 C. From Figure 3b, it is calculated that the first de-intercalation capacity and irreversible capacity loss (ICL) of PVG is about 384 mAh·g^−1^ and 64 mAh·g^−1^, with the first columbic efficiency about 86%, demonstrating its excellent reversibility. ICL is caused by the formation of solid electrolyte interface (SEI) film, which is preliminarily formed in the first cycle and matured in the subsequent cycles. In addition, the reactions of Li ions with the surface functional groups might also lead to the increase of capacity loss at the first cycle [29,30].

Figure 3c shows the capacity retention during 55 charge/discharge cycles at 1 C. In general, a three-stage capacity variation can be observed. The discharge capacity decreases rapidly in the first five cycles due to the formation of unstable SEI film. It then begins to increase continuously to 260 mAh·g^−1^ until the 12th cycle, and almost keeps unchanged during subsequent cycles, which is also found in the cycle performance of microcrystalline graphite as the anode of LIB [31]. We believe this is caused by the activation process of the electrode. Further, the result of electrochemical cycling test of PVG agrees well with the capacity fading at 1 C in Figure 3a. To further investigate the electrochemical performance of PVG, electrochemical impedance spectra (EIS) measurements were carried out over a frequency range from 10 mHz to 100 KHz, with a signal amplitude of 5 mV. Figure 3a shows the Nyquist plots of the impedance spectra for PVG half-cell before and after cycles. As for the freshly fabricated cell, there is a single depressed semicircle in the high and middle frequency regions, and an inclined line at the low frequency. The semicircle might be related to the contact resistance of electrolyte/electrode (R_s_) and charge-transfer resistance (R_ct_), and the low frequency straight line is associated with the diffusion impedance of electrolyte ions. However, the impedance spectra of PVG half-cell after 55 cycles reveals another depressed semicircle in the high frequency region, which is assigned to the impedance of SEI film formed on the graphite (R_f_). Taking into account that lithium ions’ consuming and capacity fading resulted from the formation of SEI film, the pre-lithiation process is carried out to guarantee the stable SEI film formation on the graphite surface as the anode of LICs. Thus, pre-lithiated PVG prepared from Sri Lanka graphite ore is expected to be a promising anode material for LICs with good rate capability, large capacity and high reversibility.

### 3.3. Electrochemical Performance of GPC in SCs

**Figure 4:**
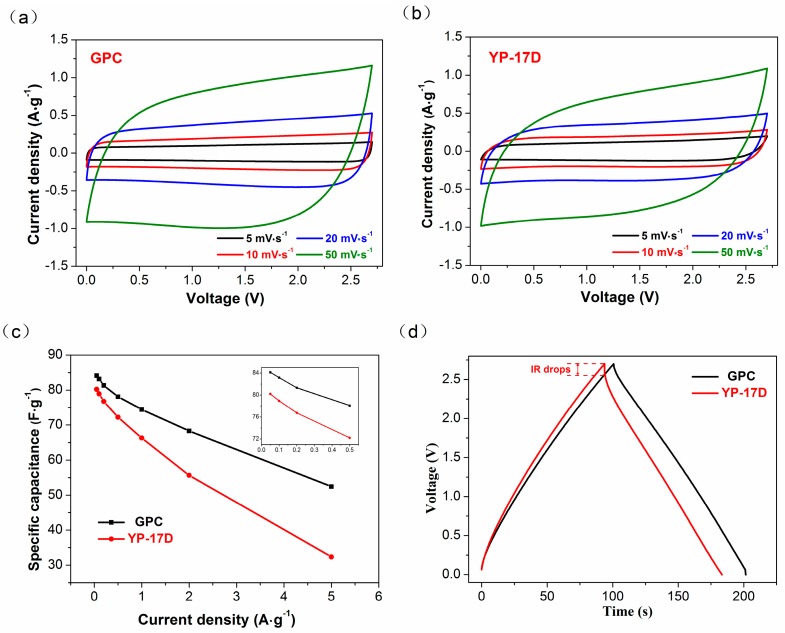
Electrochemical performances of symmetric supercapacitors, based on GPC and YP-17D with working voltage ranging from 0 to 2.7 V. (**a**) Cycle voltammetry (CV) curves of GPC at scan rates from 5 to 50 mV·s^−1^; (**b**) CV curves of YP-17D at scan rates from 5 to 50 mV·s^−1^; (**c**) Rate capability plots of SCs based on GPC and YP-17D; (**d**) galvanostatic charge–discharge (GCD) profiles of GPC and YP-17D at a current density of 1 A·g^−1^.

The electrochemical performance of GPC and YP-17D are evaluated by assembling symmetric supercapacitors. CV and galvanostatic charge/discharge cycling (GC) tests were performed within the potential window, from 0 to 2.7 V. The CV curves of GPC in Figure 4a exhibits nearly symmetric rectangular shape when the scan rate is below 20 mV·s^−1^, but shows a slight distortion when the scan rate was increased to 50 mV·s^−1^. YP-17D shows a similar curve shape in Figure 4b, with that of GPC. None of faradic peaks were examined for both CV curves, indicating an ideal electrostatic adsorption behavior and fast electrochemical response. Moreover, the CV curves of GPC are more inclined to take on a rectangular shape at high scan rates than those of YP-17D, implying the better high-rate capacitive performance in GPC. This is ascribed to its developed mesoporous structure and better electric conductivity.

Figure 4c shows the curves of specific capacitances of GPC and YP-17D as the function of the current density. GPC delivers a good rate performance with a specific capacitance of 83 F·g^−1^ at 0.05 A·g^−1^, and 64% capacitance retention when the current density was increased to 5 A·g^−1^. It can be seen that GPC displays a higher specific capacitance and better rate ability than YP-17D in all current densities. The former is attributed to the difference in porous structure and utilization of micropores while the latter is due to the enhanced electric conductivity caused by high graphitization degree of GPC. The porosity is introduced by KOH activation which facilitates the fast electrolyte transportation to micropores, and thus improves ion adsorption storage capacity. The random oriented graphitic layers form conductive networks, which reduce the bulk resistance significantly.

Figure 4d compares the galvanostatic charge–discharge (GCD) profiles of GPC and YP-17D at the current density of 1 A·g^−1^. All of the charge-discharge lines are almost straight and symmetrical, indicating the ideal electrical double-layer capacitance (EDLC) characteristics with an excellent reversibility. Generally, IR drops is used to evaluate electrochemical resistance of electrodes. There is no obvious IR drop in the GCD lines of GPC, while an IR drop of 0.1 V can be seen in that of YP-17D. This again confirms the smaller electrochemical resistance, and thus faster electrochemical response speed, in GPC electrode. The characteristics of GCD profiles further confirm the results from CV tests, and specific capacitance function with current density. In summary, GPC is a promising cathode material for SCs because of its relatively ideal EDLC electrochemical response, notable specific capacitance, and excellent rate performance.

### 3.4. Electrochemical Performance of GPC in LICs

**Figure 5:**
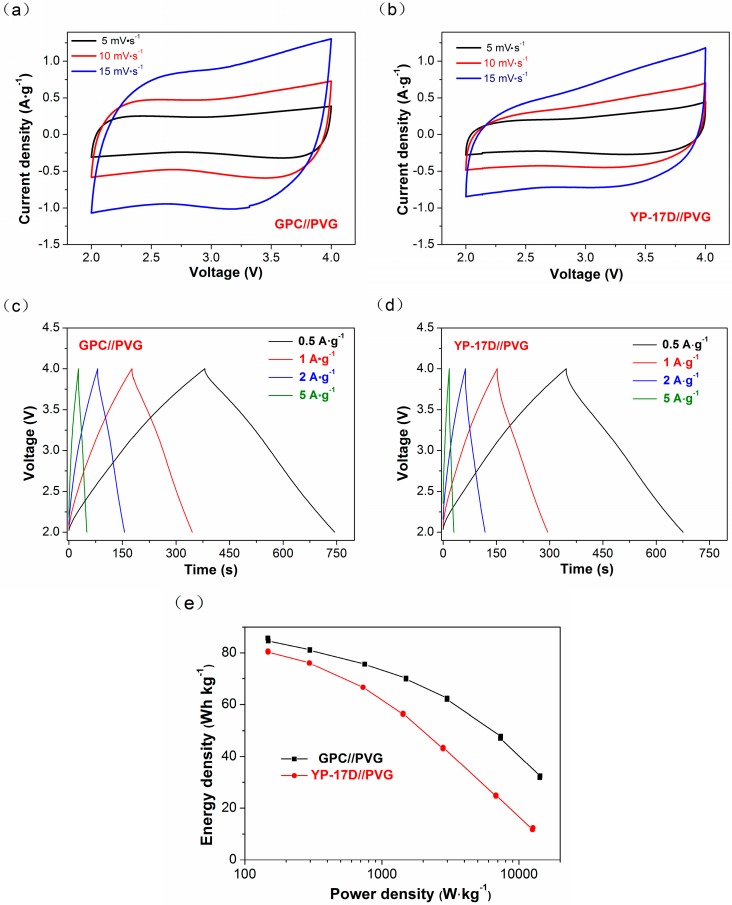
Electrochemical performances of hybrid devices fabricated with different cathodes. (**a**) CV curves of GPC//PVG; (**b**) CV curves of YP-17D//PVG; (**c**) GCD profiles of GPC//PVG at different current densities; (**d**) GCD profiles of YP-17D//PVG at different current densities; (**e**) Ragone plots of GPC//PVG and YP-17D//PVG.

**Figure A1:**
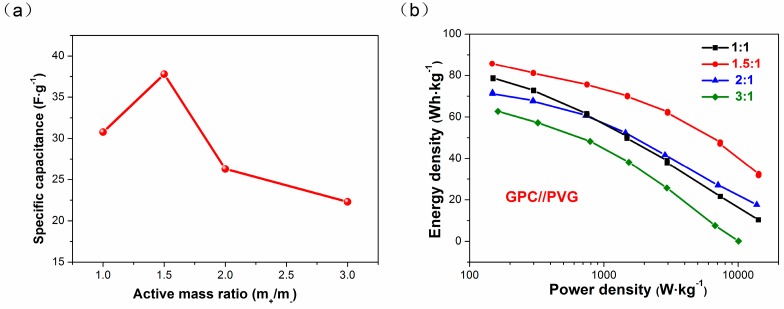
Electrochemical performance of the GPC//PVG devices assembled with different mass ratio of electrodes. (**a**) The capacity performance of LICs at 1 A·g^−1^; (**b**) Ragone plots.

As mentioned above, multi-structure GPC is a potential replacement of YP-17D for its extraordinary capacitance property. We further investigate its electrochemical performances by fabricating hybrid LICs of GPC//PVG and YP-17D//PVG, based on GPC and YP-17D cathodes, respectively. Pre-lithiated PVG is used as the anode in all LICs devices. Optimizing device performance consists of achieving the suitable working voltage window and balancing the active mass of electrodes. Our previous investigation found that the galvanostatic charge/discharge profile of graphitized porous carbon (GPC) exhibited a line curve with the potential window from 2 to 4.5 V (vs. Li^+^/Li) in a Li half-cell system [11]. As such, a safe working voltage region was set as 2–4 V, so as to maintain the cathode operating in the ion surface adsorption regime, while limiting ion insertion. The mass ratio of electrodes can often be well-established based on the principle of balanced charge passing through the cathode and the anode (Q_+_/Q_−_). According to the results of specific capacity of PVG half-cell and GPC symmetric cell, the typical capacity of GPC cathode was selected as 75 F·g^−1^ (56 mAh·g^−1^) at 1 A·g^−1^, while the PVG anode revealed a discharge capacity of 67 mAh·g^−1^ at 5 C (C is defined based on the capacity of graphite theory). Thus, the theoretical mass ratio of the LIC was determined to be 1.16. To further confirm the mass ratio of positive to negative electrode, we measured the capacity of LICs assembled with different mass ratio (m_+_/m_−_) of GPC and PVG ranging from 1 to 4, as shown in Figure A1a,b. The maximum specific capacity of the LICs appears when the value of mass ratio reaches 1.5, which is higher than the above predicted value. Meanwhile, GPC//PVG device exhibits the best energy and power performance at the mass ratio of 1.5. Therefore, the electrochemical performance of GPC//PVG LIC was investigated at the voltage window of 2–4 V, with an optimal mass ratio of 1.5. As shown in Figure 5a, nearly symmetric rectangular CV curves are observed in GPC//PVG at a low scan rate (5, 10 and 15 mV·s^−1^). Compared with CV curves of the supercapacitor (Figure 5b), there exist slight distortions ascribed to the lithium ion intercalation/de-intercalation on graphite electrode. A more severe distortion observed in YP-17D//PVG at the same scan rate indicates the fast electrochemical response of GPC, coupling well with the result in Figure 4a. This is attributed to the high specific area and hierarchical porous structure of GPC. Moreover, the CV curves distorted greatly from rectangular shape in the hybrid devices, similar to the CV results of the SCs. It is worth noting that the microstructure of cathode materials could significantly affect the capacitive behavior. A more developed porous structure, and better electric conductivity, contribute to a faster electrochemical response of the hybrid devices. Therefore, GPC is further proved to be a favorable electrode material for hybrid devices. The GPC//PVG hybrid device shows a much better capacitive performance than that of YP-17D//PVG device.

GCD curves of GPC//PVG and YP-17D//PVG hybrid systems at different current densities (0.5 A·g^−1^, 1 A·g^−1^, 2 A·g^−1^ and 5 A·g^−1^) are shown in Figure 5c,d. GPC//PVG reveals an almost straight and symmetrical triangular shape in the voltage window of 2–4 V, indicating its dominant EDLC behavior with excellent reversibility. Further, none of faradic charge storage feature is displayed because lithium intercalation/de-intercalation reaction takes place below 1.5 V. The major deference between GCD curves of GPC//PVG and YP-17D//PVG is the discharge time and IR drops value. Normally, the discharge time is closely related to a specific capacity, while the IR drops are used to evaluate electrochemical conductivity of electrodes. Compared to the 0.45 V IR drops in YP-17D//PVG at the current density of 5 A·g^−1^, there are only IR drops of 0.35 V to be observed in GPC//PVG at the same condition. This result, agreeing with GCD characteristics of SCs, is ascribed to the smaller ion diffusion resistance of GPC caused by the presence of a conductive graphitic structure.

For further investigation of the electrochemical performance of GPC in the hybrid systems, Ragone plots were plotted based on the total mass of the active material in both electrodes, as shown in Figure 5d. The GPC//PVG system delivers a high-energy density of 86 W·h·kg^−1^ at 150 W·kg^−1^ and still maintains 48 W·h·kg^−1^ at 7.4 kW·kg^−1^. This suggests its prominent rate performance, with 55.8% retention at an extremely high power density. The YP-17D//PVG LIC displays a relatively lower energy density of 80 W·h·kg^−1^ at 147 W·kg^−1^, and a rapid decline at high power densities. Further, the performance gap enlarges gradually with the increase of charge/discharge densities. These demonstrate the excellent high-power performance of GPC//PVG system. It is believed that the energy density mainly depends on the EDLC capacitive performance of cathode, while the powder density is mainly determined by the lithium insertion/extraction properties of anode. Such an excellent electrochemical performance of GPC//PVG LIC is attributed to the following reasons: (a) good electronic conductivity of GPC cathode due to the graphitic carbon in the amorphous porous carbon; and (b) smooth lithium ion diffusion channels resulting from a large specific surface area, and favorable mesoporous structure.

## 4. Conclusions

In this work, starting from Sri Lanka graphite ore, graphitic porous carbon and high-purity vein graphite have been prepared and separately employed as cathode and anode material for LIC. The assembled LIC hybrid device delivers a maximum energy density of 86 W·h·kg^−1^ at 150 W·kg^−1^, and 55.8% retention at 7.4 kW·kg^−1^. This good electrochemical performance is attributed to the developed porous structure which can facilitate a fast electrolyte transportation, and promotes the full utilization of micropores, as well as excellent electric conductivity.

## References

[1] Ren G., Ma G., Cong N. (2015). Review of electrical energy storage system for vehicular applications. Renew. Sustain. Energy Rev..

[2] Choi N.S., Chen Z.H., Freunberger S.A., Ji X.L., Sun Y.K., Amine K., Yushin G., Nazar L.F., Cho J., Bruce P.G. (2012). Challenges facing lithium batteries and electrical double-layer capacitors. Angew. Chem. Int. Ed..

[3] Ji L., Meduri P., Agubra V., Xiao X., Alcoutlabi M. (2016). Graphene-based nanocomposites for energy storage. Adv. Energy Mater..

[4] Gu H.C., Zhu Y.E., Yang J.Q., Wei J.P., Zhou Z. (2016). Nanomaterials and technologies for lithium-ion hybrid supercapacitors. Chemnanomat.

[5] Wen L., Liu C.M., Song R.S., Luo H.Z., Shi Y., Li F., Cheng H.M. (2014). Lithium storage characteristics and possible applications of graphene materials. Acta Chim. Sin..

[6] Ma Y., Chang H., Zhang M., Chen Y. (2015). Graphene-based materials for lithium-ion hybrid supercapacitors. Adv. Mater..

[7] Yang J.J., Kim Y.R., Jeong M.G., Yuk Y.J., Kim H.J., Park S.G. (2015). Synthesis and electrochemical characteristics of spherical Li_4_Ti_5_O_12_/CNT composite materials for hybrid capacitors. J. Electrochem. Sci. Technol..

[8] Han X., Han P., Yao J., Zhang S., Cao X., Xiong J., Zhang J., Cui G. (2016). Nitrogen-doped carbonized polyimide microsphere as a novel anode material for high performance lithium ion capacitors. Electrochim. Acta.

[9] Yu X., Zhan C., Lv R., Bai Y., Lin Y., Huang Z.-H., Shen W., Qiu X., Kang F. (2015). Ultrahigh-rate and high-density lithium-ion capacitors through hybriding nitrogen-enriched hierarchical porous carbon cathode with prelithiated microcrystalline graphite anode. Nano Energy.

[10] Frackowiak E., Beguin F. (2001). Carbon materials for the electrochemical storage of energy in capacitors. Carbon.

[11] Lei Y., Huang Z.H., Yang Y., Shen W.C., Zheng Y.P., Sun H.Y., Kang F.Y. (2013). Porous mesocarbon microbeads with graphitic shells: Constructing a high-rate, high-capacity cathode for hybrid supercapacitor. Sci. Rep. UK.

[12] Zhang J., Liu X.F., Wang J., Shi J.L., Shi Z.Q. (2016). Different types of pre-lithiated hard carbon as negative electrode material for lithium-ion capacitors. Electrochim. Acta.

[13] Khomenko V., Raymundo-Piñero E., Béguin F. (2008). High-energy density graphite/ac capacitor in organic electrolyte. J. Power Sources.

[14] Wang H., Yoshio M. (2008). Performance of ac/graphite capacitors at high weight ratios of AC/graphite. J. Power Sources.

[15] Zhang J., Shi Z., Wang J., Shi J. (2015). Composite of mesocarbon microbeads/hard carbon as anode material for lithium ion capacitor with high electrochemical performance. J. Electroanal. Chem..

[16] Zhang J., Shi Z., Wang C. (2014). Effect of pre-lithiation degrees of mesocarbon microbeads anode on the electrochemical performance of lithium-ion capacitors. Electrochim. Acta.

[17] Wang H.W., Guan C., Wang X.F., Fan H.J. (2015). A high energy and power Li-ion capacitor based on a TiO_2_ nanobelt array anode and a graphene hydrogel cathode. Small.

[18] Fan Z.J., Yan J., Wei T., Zhi L.J., Ning G.Q., Li T.Y., Wei F. (2011). Asymmetric supercapacitors based on graphene/MnO_2_ and activated carbon nanofiber electrodes with high power and energy density. Adv. Funct. Mater..

[19] Ma S.B., Nam K.W., Yoon W.S., Yang X.Q., Ahn K.Y., Oh K.H., Kim K.B. (2007). A novel concept of hybrid capacitor based on manganese oxide materials. Electrochem. Commun..

[20] Luo L., Tan X., Tian J. (2007). Research progress of graphite purification. Non-Met. Min..

[21] Puthusseri D., Aravindan V., Madhavi S., Ogale S. (2014). Improving the energy density of li-ion capacitors using polymer-derived porous carbons as cathode. Electrochim. Acta.

[22] Pramanik A., Maiti S., Mahanty S. (2015). Reduced graphene oxide anchored Cu(OH)_2_ as a high performance electrochemical supercapacitor. Dalton Trans..

[23] Shi Z.Q., Zhang J., Wang J., Shi J.L., Wang C.Y. (2015). Effect of the capacity design of activated carbon cathode on the electrochemical performance of lithium-ion capacitors. Electrochim. Acta.

[24] Ye L., Liang Q., Lei Y., Yu X., Han C., Shen W., Huang Z.-H., Kang F., Yang Q.-H. (2015). A high performance li-ion capacitor constructed with Li_4_Ti_5_O_12_/c hybrid and porous graphene macroform. J. Power Sources.

[25] Tu F.Y., Liu S.Q., Wu T.H., Jin G.H., Pan C.Y. (2014). Porous graphene as cathode material for lithium ion capacitor with high electrochemical performance. Powder Technol..

[26] Lu L., Sahajwalla V., Kong C., Harris D. (2001). Quantitative X-ray diffraction analysis and its application to various coals. Carbon.

[27] Lillo-Rodenas M.A., Cazorla-Amoros D., Linares-Solano A. (2003). Understanding chemical reactions between carbons and NaOH and KOH: An insight into the chemical activation mechanism. Carbon.

[28] Lillo-Rodenas M.A., Juan-Juan J., Cazorla-Amoros D., Linares-Solano A. (2004). About reactions occurring during chemical activation with hydroxides. Carbon.

[29] Agubra V.A., Zuniga L., Flores D., Villareal J., Alcoutlabi M. (2016). Composite nanofibers as advanced materials for Li-ion, Li-O_2_ and Li-S batteries. Electrochim. Acta.

[30] Ji L.W., Lin Z., Alcoutlabi M., Zhang X.W. (2011). Recent developments in nanostructured anode materials for rechargeable lithium-ion batteries. Energy Environ. Sci..

[31] Zou L., Kang F., Li X., Zheng Y.-P., Shen W., Zhang J. (2008). Investigations on the modified natural graphite as anode materials in lithium ion battery. J. Phys. Chem. Solids.

